# Phosphorus Availability Alters the Effects of Silver Nanoparticles on Periphyton Growth and Stoichiometry

**DOI:** 10.1371/journal.pone.0129328

**Published:** 2015-06-15

**Authors:** Beth C. Norman, Marguerite A. Xenopoulos, Daniel Braun, Paul C. Frost

**Affiliations:** Department of Biology, Trent University, Peterborough, Ontario, Canada; VIT University, INDIA

## Abstract

Exposure to silver nanoparticles (AgNPs) may alter the structure and function of freshwater ecosystems. However, there remains a paucity of studies investigating the effects of AgNP exposure on freshwater communities in the natural environment where interactions with the ambient environment may modify AgNP toxicity. We used nutrient diffusing substrates to determine the interactive effects of AgNP exposure and phosphorus (P) enrichment on natural assemblages of periphyton in three Canadian Shield lakes. The lakes were all phosphorus poor and spanned a gradient of dissolved organic carbon availability. Ag slowly accumulated in the exposed periphyton, which decreased periphyton carbon and chlorophyll *a* content and increased periphyton C:P and N:P in the carbon rich lakes. We found significant interactions between AgNP and P treatments on periphyton carbon, autotroph standing crop and periphyton stoichiometry in the carbon poor lake such that P enhanced the negative effects of AgNPs on chlorophyll *a* and lessened the impact of AgNP exposure on periphyton stoichiometry. Our results contrast with those of other studies demonstrating that P addition decreases metal toxicity for phytoplankton, suggesting that benthic and pelagic primary producers may react differently to AgNP exposure and highlighting the importance of *in situ* assays when assessing potential effects of AgNPs in fresh waters.

## Introduction

Nanotechnology is a growing and increasingly pervasive industry. The use of silver nanoparticles (AgNPs) is particularly prevalent due to their antimicrobial properties. These antimicrobial properties result partly from the release of ionic silver from the particulate form [1] and partly from interactions of the nanoparticles with cellular membranes and internal cellular structures [2]. Given the growing use of AgNPs in consumer products and the probability of their release into the environment [3], there continues to be a need to understand their effects on organisms and ecological interactions.

AgNPs leached from consumer products can enter freshwaters via several pathways, including treated or untreated waste water discharge and runoff from terrestrial applications of waste water treatment sludge [4]. Under laboratory conditions, AgNP exposure can depress the abundance and growth of heterotrophic microbes [5,6] and algae [7]. AgNPs also appear to affect microbe-driven processes such as primary production [1], enzyme activity [8,9], ammonia oxidation [10,11] and organic matter decomposition [12]. Significant changes in these microbial processes resulting from AgNP exposure could have far-reaching consequences for aquatic ecosystems including altered community composition [9], ecosystem metabolism [13] and nutrient cycling [14].

A full assessment of the consequences of AgNP exposure for aquatic ecosystems will need to include the complex ecological and physico-chemical context of natural environments. This realism is lacking in laboratory-scale studies where single species are often exposed to AgNPs under controlled and simplified environmental conditions. One important aspect of the environmental template in aquatic ecosystems is the supply of limiting nutrients such as nitrogen (N) and phosphorus (P). Increased supplies of N or P can affect community and elemental composition of primary producers [15,16], trophic dynamics [17], and ecosystem function [18,19]. Nutrient availability can also modify the uptake and toxicity of dissolved metals for freshwater organisms. For example, Riedel and Sanders [20] found that the increased phytoplankton biomass resulting from nutrient addition led to greater uptake of cadmium, nickel, and zinc from the water column in estuaries. Phosphorus-rich algae may be less susceptible to metal toxicity due to metal sequestration by intracellular polyphosphates ([21] and citations within). Although relatively few studies have investigated the influence of eutrophication on AgNP toxicity, it appears that increased levels of nutrient availability may lessen the negative effects of AgNP exposure on aquatic bacterial [22], algal [23], zooplankton [24] and fungal communities [25]. Environmental phosphate may also serve as a natural ligand for silver ions released from AgNPs and further reduce an organism’s toxic response [22,24].

In aquatic ecosystems, AgNPs are likely to aggregate and settle out of the water column [26], perhaps disproportionally increasing exposure to benthic organisms. Despite this, most studies have focused on planktonic or otherwise open water organisms. Here, we investigated the interacting effects of AgNP exposure and P availability on lentic periphyton. We used diffusing substrates to expose periphyton to AgNPs under increasing levels of P enrichment. These assays have been recommended for improving our understanding of the effects of novel contaminants [27]. We measured the main and interactive effects of AgNP exposure and P availability on periphyton biomass and stoichiometry to determine how the nutritional context would affect the toxicity of this emerging contaminant under natural lake environmental conditions.

## Materials and Methods

### Study sites

We completed our experiments at the Experimental Lakes Area (ELA) in northwestern Ontario, Canada (access to site granted by the Ontario Ministry of Natural Resources and Forestry). We placed nutrient diffusing substrates [28] in three separate lakes (L224, L239, and L222) in the summer of 2012. The chemical and physical characteristics of the lakes, including temperature, light penetration, dissolved organic carbon (DOC), total dissolved nitrogen (TDN), and total dissolved phosphorus (TDP) were measured every three weeks during the summer of 2012 (end of May-middle of Aug). These lakes span a gradient of DOC availability with lowest values (~4 mg C/L) in L224 and the highest values (~10.5 mg C/L) in L222 (Table 1). All three lakes are relatively phosphorus poor as is typical for lakes in this region [15,29].

**Table 1:** Mean nutrient content, light extinction coefficients, and daily temperature (standard deviation) measured periodically June-August 2012.

Lake	DOC (mg/L)		TDN (μg/L)		TDP (μg/L)		Light extinction coefficient (/m)		Temperature (°C)	
**L224**	4.21	(0.44)	195.07	(13.13)	6.10	(1.02)	0.254	(0.063)	22.5	(2.15)
**L239**	8.56	(2.37)	206.20	(18.59)	7.28	(2.82)	0.627	(0.106)	22.3	(2.20)
**L222**	10.47	(0.57)	356.18	(38.99)	7.04	(1.21)	0.810	(0.158)	23.3	(2.18)

Nutrient samples and light profile data were collected from the epilimnion at the deepest part of the lake (n = 5). Temperature was recorded every 30 min at 1 m depth for L222 and L224 and every hour at the surface for L239 using data loggers.

### Experimental design

We used nutrient diffusing substrates to grow periphyton that was exposed to different AgNP and P environments. We had three levels of each factor (AgNPs and P), which created nine treatment combinations. We built three replicate substrates for each combination per lake. Substrates were placed into lakes for about 1 month (17 July-12, 13, 14 Aug 2012) at a depth of approximately 0.5 m.

Substrates consisted of unglazed, terra cotta clay pots filled with 5% agar solutions amended with 0.035 M total N (added as NH_4_NO_3_) to prevent N limitation. To manipulate the P environment, we added KH_2_PO_4_ to the agar to achieve the following PO_4_-P concentrations: 0 mM (No-P), 0.5 mM (Low-P), and 5 mM (High-P). This high concentration of P was chosen as it was previously found to greatly reduce P-limitation of periphyton in these lakes [15]. To manipulate AgNP exposure, we added spherical AgNPs capped with polyvinylpyrollidone (PVP) to create the following Ag concentrations: 0 mg/L (No-Ag), 3 mg/L (Low-Ag), and 12 mg/L (High-Ag). Although these silver concentrations are greater than those predicted for surface waters [30], we assumed that ionic or particulate silver would slowly diffuse through the clay [27], exposing periphyton to lower AgNP doses than what we added to the agar. In addition, benthic environments are likely to experience higher concentrations of AgNPs due to sedimentation [31]. After agar solutions were poured into each substrate and allowed to set, petri plates were sealed over the pot openings with non-toxic silicon, and the substrates were attached to wire baskets. Baskets were placed in each lake so that the substrates were approximately 0.5 m deep.

### AgNP Characterization

The AgNP solution used was manufactured by NanoComposix (San Diego, CA) with a nominal concentration of 1 mg Ag/mL suspended in Milli-Q water. Nanoparticles were spherically shaped with an average diameter of 50 nm. Upon receipt (June 2012), the average hydrodynamic diameter of the particles was confirmed as 56.3 using dynamic light scattering (DLS) and the stock solution concentration was measured as 0.96 mg Ag/mL using inductively coupled plasma mass spectrometry (ICP-MS) following an acid digestion [26]. The stock solution contained a small percentage (<3%) of dissolved ionic silver [26].

We used the same AgNP stock solution in concurrent *in situ* experiments in L239 and extensively characterized its fate and behavior in the water. These AgNPs have a relatively long residence time in L239 water (half life ~20 d), following an initial period of rapid dissipation, and form hetero-agglomerates in the L239 matrix [26]. Very little ionic Ag was detected in L239 water [26], and MINEQL^+^ modelling suggests that most Ag^+^ would complex with dissolved organic matter (unpublished data) in this humic-rich lake.

### Periphyton collection and analysis

On the day of sampling, we carefully removed each substrate from the baskets and gently scrubbed them with a soft-bristled toothbrush. We rinsed all material from each substrate into individual plastic dishes with lake water. These periphyton slurries were returned to the lab in sealed plastic bags that were held in the dark. We gently homogenized each sample with one or two pulses of a blender (set to low) prior to subsampling.

We filtered two subsamples of the resulting slurry onto 0.7 μm glass fiber filters (Whatman GF/F) for subsequent measurement of chlorophyll *a* (Chl-*a*). Filters were kept frozen in the dark until they were analyzed using a cold ethanol extraction [32] and Chl-*a* content was quantified using a fluorometer (Varian Cary Eclipse). Additional slurry subsamples were filtered onto ashed 0.7 μm glass fiber filters (Whatman GF/F) and dried at 60°C for elemental analysis. On two of the filters, we measured periphyton C and N content using a CN analyzer (Vario EL, Elementar). P content was determined from the remaining two filters via the molybdate-blue colormetric method and spectrophotometry (UV-Visible Spectrophotometer, 50 Bio) following an alkaline persulfate digestion [33].

A final slurry subsample was filtered onto a 0.8 μm polycarbonate membrane. Membranes were preserved in 4% nitric acid and digested on a heat block at 70°C for 2 hours. Filters were removed and the solutions were re-filtered and analyzed for Ag content using ICP-MS (Thermo Scientific, model X Series 2, Nepean, ON, Canada). Standard solutions were prepared from a stock solution of 1000 mg Ag/L in 2% nitric acid and Milli-Q water. The quadrupole was operated in peak hopping scan mode monitoring ^107^Ag with an internal standard of indium (^115^In) in dwell time of 10 s [23]. Ag values that were below detection (<0.006 μg/L) were replaced with a zero value to calculate mean periphyton Ag content.

### Statistical analysis

Differences among treatments with in each lake were determined using 2-way analysis of variance (ANOVA). When there was a significant P*Ag interaction, Tukey post-hoc comparisons were used to determine pairwise differences among AgNP treatments within the same P treatment. All *p*-values presented in the text were generated from these pairwise comparisons while the main and interactive effects are shown in figure panels. All ANOVA response variables, with the exception of periphyton Chl-*a* content, were natural logarithm-transformed to meet assumptions of homoscedasticity. Two-tailed t-tests were used to evaluate differences between mean Chl-*a* content of periphyton collected from Low-Ag and High-Ag substrates within the same P treatment. All statistical analyses were performed using SigmaPlot with SigmaStat integration (v. 12.0).

## Results

Periphyton Ag content increased with greater Ag concentrations in the agar (with the exception of L222; Table 2), although differences among Ag treatments within the same lake were generally not significant. However, we found low levels of Ag even on No-Ag substrates and there was some variation in Ag content even among substrates in the same treatment (Table 2). All values were especially low (total Ag ~1–100 ng on entire substrate) given the substantial quantity (total Ag ~2.0 mg in the High Ag treatment) of AgNPs added to the agar at the beginning of the experiment. More Ag also appeared to accumulate on substrates incubated in L222 than L239 or L224 at all levels of AgNP exposure.

**Table 2:** Mean (range) Ag content (ng Ag/cm^2^) of periphyton collected from all three lakes.

	L224		L239		L222	
***No P***						
** No Ag**	0.012^a^	(0.005–0.020)	0.035a	(0.016–0.054)	0.209^a^	(0.079–0.435)
** Low Ag**	0.350^a^	(0.080–0.817)	0.114^a^	(0.037–0.167)	0.804^a^	(0.308–1.226)
** High Ag**	0.731^a^	(0.022–2.138)	3.95^a^	(0.030–7.870)	1.59^a^	(0.0923–0.773)
***Low P***						
** No Ag**	0.020^a^	(bd-0.040)	0.014^a^	(bd-0.024)	0.206^a^	(0.066–0.449)
** Low Ag**	0.159^a^	(bd-0.391)	0.067^a^	(0.013–0.157)	7.01^b^	(0.105–12.644)
** High Ag**	0.179^a^	(0.017–0.469)	0.290^a^	(0.090–0.581)	0.337^a^	(0.092–0.773)
***High P***						
** No Ag**	0.033^a^	(bd-0.059)	0^a^	(bd)	0.109^a^	(0.035–0.182)
** Low Ag**	0.274^a^	(0.016–0.549)	0.013^a^	(bd-0.025)	1.85^a^	(0.258–4.753)
** High Ag**	0.371^a^	(0.122–0.499)	0.038^a^	(bd-0.076)	0.874^a^	(0.285–1.613)

Values are averages of three replicate substrates. bd indicates Ag measurements that were below detection. Means within the same lake with different lowercase letters are significantly different (*p* < 0.05).

**Fig 1:**
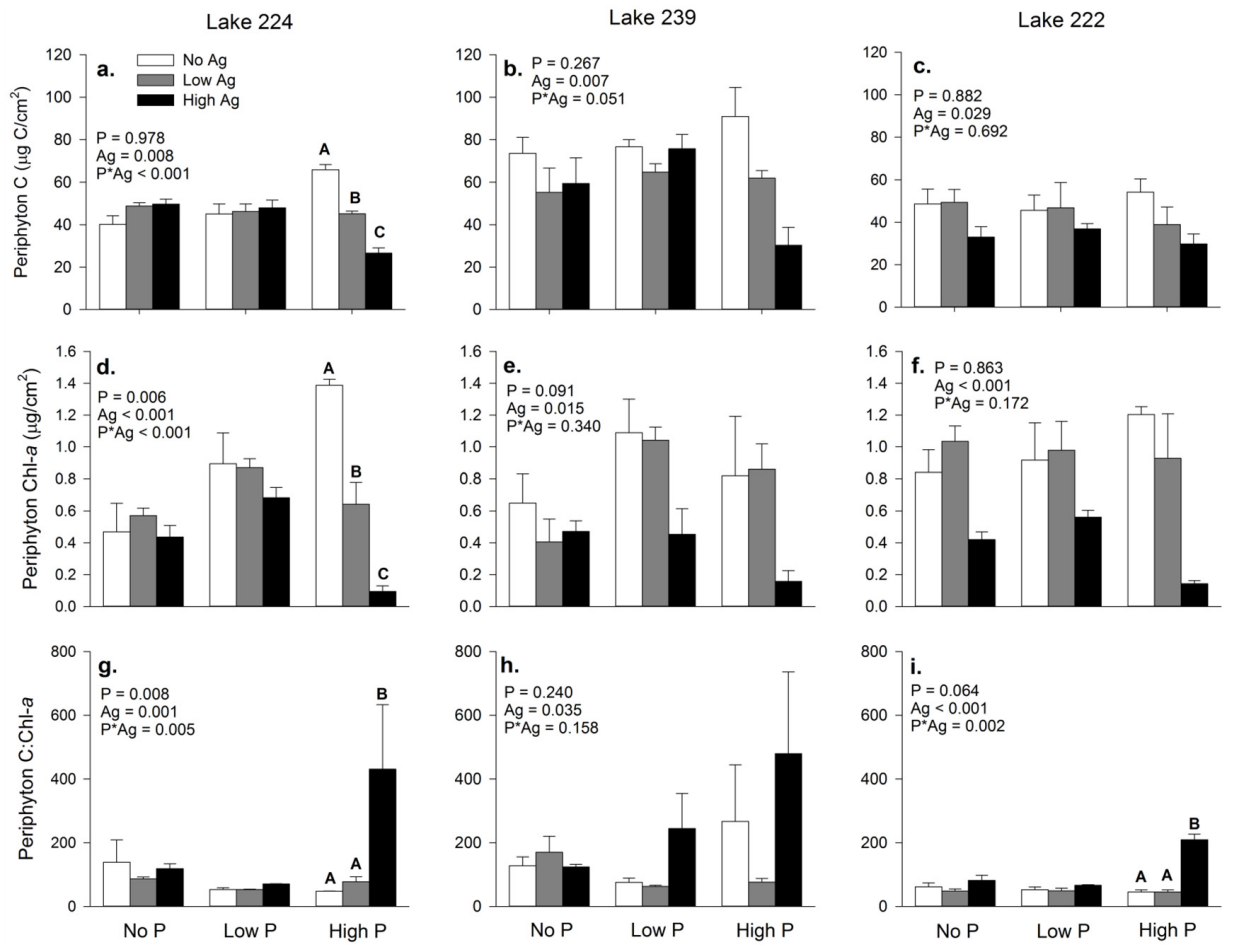
Periphyton C, Chl-*a* and autotrophic index from all three lakes. Bars are means of three substrates with standard error whiskers. *p* values for main and interactive effects (2-way ANOVA) are presented in each panel. For variables with a significant P*Ag interaction, different capital letters indicate significantly different means among AgNP treatments within the same P treatment as determined by Tukey pairwise comparisons.

AgNP exposure had significant effects on periphyton biomass and autotroph standing crop in L224 but these effects were different depending on the level of P enrichment (Fig 1a and 1d). Under No-P and Low-P conditions in this lake, periphyton C and Chl-*a* were similar among all AgNP treatments. However, under High-P conditions, periphyton C on substrates with added AgNPs was 70–40% of that in periphyton from the No-Ag substrates (Tukey pairwise comparison, *p* ≤ 0.001). Strikingly, Chl-*a* concentrations on the Low-Ag and High-Ag substrates were < 50% and < 10% that of the No-Ag substrates, respectively (Tukey pairwise comparison, *p* < 0.006). In L239 and L222, both C content and autotroph standing crop was lower on substrates exposed to AgNPs. Phosphorus addition generally appeared to enhance these effects in these lakes, although not significantly so (Fig 1b, 1c, 1e and 1f).

The effect of AgNP exposure on C:Chl-*a* ratios in the periphyton depended on the level of P enrichment for L224 and L222. AgNP exposure significantly increased the C:Chl-*a* under High-P conditions but had no effect on C:Chl-*a* under No-P or Low-P conditions in both lakes (Fig 1g and 1i). In L239, the C:Chl-*a* of periphyton collected from substrates with AgNPs added was greater than those without AgNPs under all P conditions (Fig 1h).

**Fig 2:**
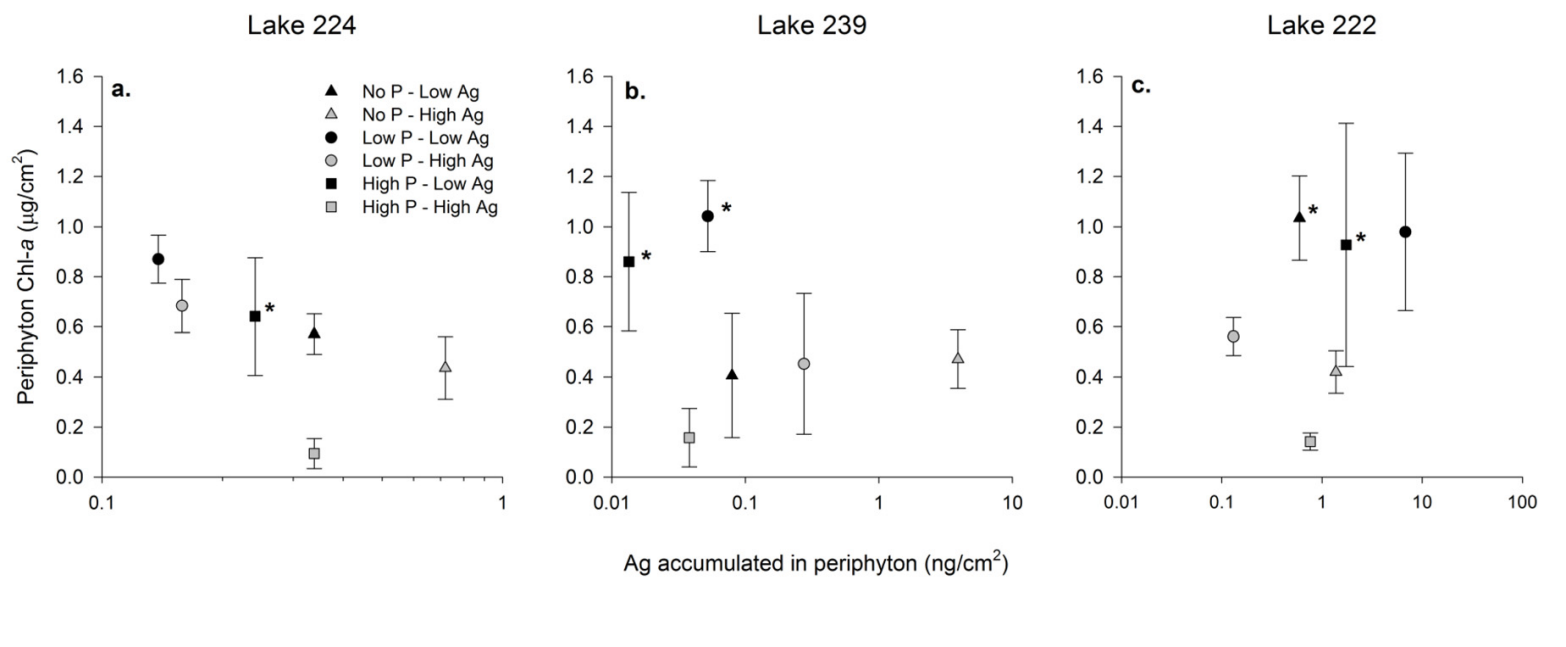
Relationship between background-corrected periphyton Ag and Chl-*a*. Background-corrected periphyton Ag was calculated as Ag content in treatments with AgNPs added—Ag in No-Ag treatment from the same P treatments in L224 (a), L239 (b), and L222 (c). Different symbols represent P treatments with Low-Ag treatments shown as filled symbols and High-Ag treatments shown as shaded symbols. Asterisks indicate low Low-Ag treatments that are significantly different than the corresponding High-Ag treatments with in the same P treatment. Error bars around symbols are standard deviations. Note the log scale for the x-axes.

We calculated the total periphyton Ag load derived from our AgNP treatments by subtracting the Ag content of periphyton collected from No-Ag substrates from the Ag content of periphyton collected from Low-Ag and High-Ag substrates with in the same P treatments for each lake. Periphyton Chl-*a* generally declined with greater Ag content in L224 (Fig 2a), although this difference was only significant under High-P conditions (t-test, *p* = 0.017). A similar pattern was found in L239 (Fig 2b), although again, differences in periphyton Chl-*a* content were only significant under conditions of P enrichment (t-test, *p* < 0.031). Results from L222 were difficult to interpret because the background-corrected Ag load in periphyton from Low-Ag substrates was greater than High-Ag substrates in both the Low-P and High-P treatments (Fig 2c).

**Fig 3:**
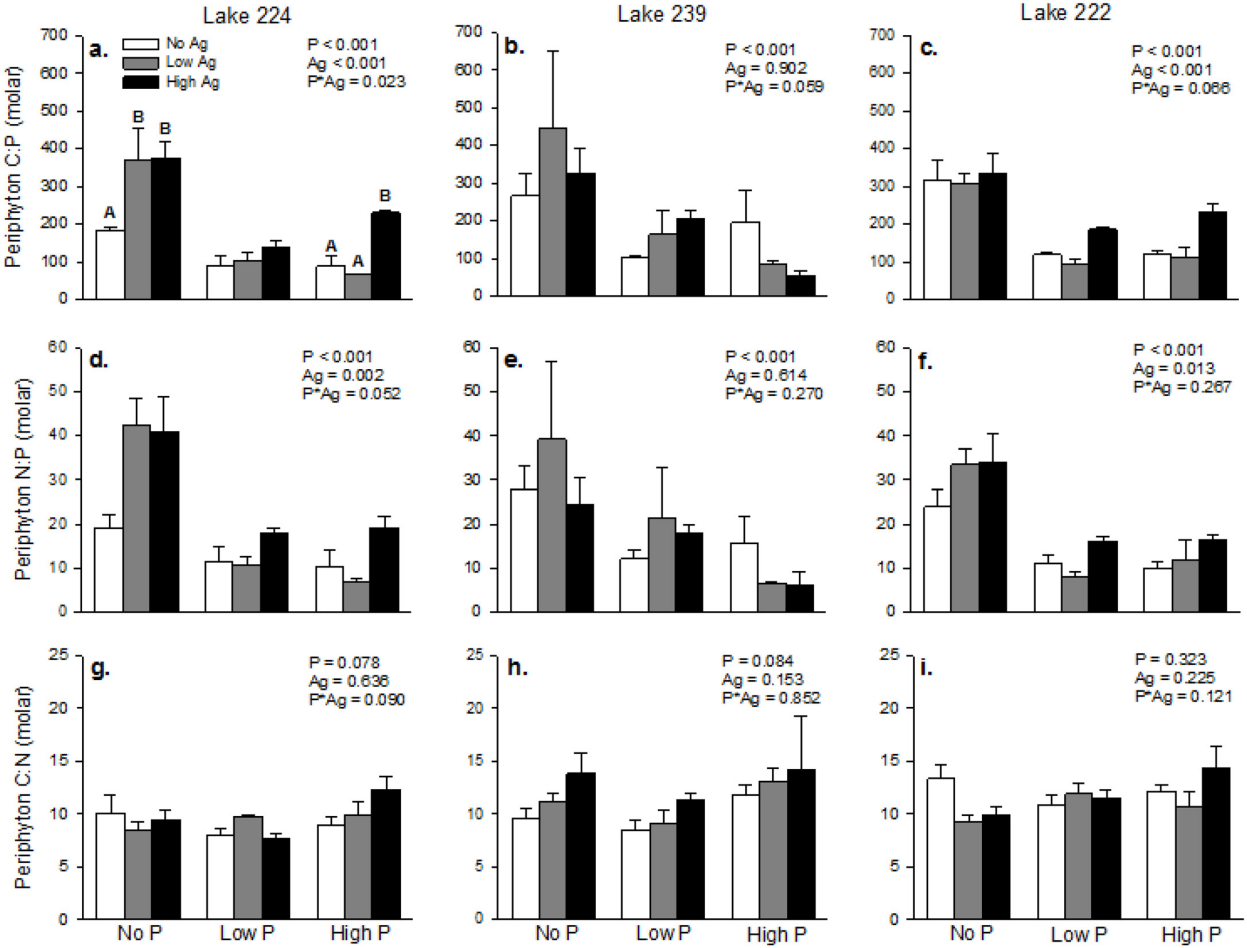
Periphyton stoichiometry from all three lakes. Bars are means of three substrates with standard deviation whiskers. P values for main and interactive effects (2-way ANOVA) are presented in each panel. For variables with a significant P*Ag interaction, different capital letters indicate significantly different means among AgNP treatments within the same P treatment as determined by Tukey pairwise comparisons.

AgNP exposure also affected periphyton stoichiometry (Fig 3). In L224, these effects depended on the level of P enrichment. In the absence of P enrichment, periphyton C:P was greater on both Low-Ag and High-Ag substrates. P enrichment dampened this effect. Periphyton C:P was similar among AgNP treatments under Low-P conditions and only the High-Ag treatment influenced periphyton C:P under the High-P conditions (Fig 3a). N:P of periphyton collected from L224 decreased in response to P enrichment and increased in response to AgNP exposure, but there was no significant interaction between the two main effects (Fig 3d).

In L239, periphyton collected from substrates with added P were more P-rich, as reflected in decreased C:P and N:P, regardless of AgNP exposure. Neither ratio responded to AgNP exposure in this lake (Fig 3b and 3e). In L222, however, AgNP exposure increased both C:P and N:P of the periphyton (Fig 3c and 3f). P enrichment resulted in periphyton with decreased C:P and N:P ratios in this lake, but there were no interactive effects between P enrichment and AgNP exposure. There were no significant interactive or main effects on periphyton C:N in any of the three lakes (Fig 3g–3i).

## Discussion

The Ag content of the periphyton generally increased along our AgNP treatment gradient, although there was considerable variation among replicates in all of the lakes. A very small quantity of Ag was detected in periphyton collected from No-Ag substrates. This may have been due to leaching from nearby substrates, Ag transfer by macroinvertebrates, or background Ag levels in the lakes. The higher Ag values recorded in periphyton from No-Ag substrates in L222 may have resulted from contamination during sample collection and processing. The use of clay pots as diffusing substrates may have contributed to the low and variable Ag content in the periphyton by interfering with AgNP and Ag^+^ transport. Although the movement of nanoparticles in soils has yet to receive thorough examination [34], soils with high clay content have been shown to be more retentive of AgNPs compared to sandy soils [35]. Clay content can also affect ionic Ag retention in soils [36]. The low amounts of Ag found in the periphyton in this study (usually <1 ng cm^-2^) may indicate that much of the Ag that we added was retained by the clay pots in either particulate or ionic form. However, the fact that we saw significant Ag effects for periphyton growth and stoichiometry suggests that our treatments were effective and that even low doses of Ag exposure may be toxic to periphyton growth.

AgNP exposure reduced autotroph standing crop in our experiment. This finding agrees with studies showing that AgNPs decrease algal growth and primary production in laboratory monocultures [1,37,38,39,40] and natural phytoplankton assemblages [23]. Depending on AgNP behavior with in the agar and diffusion across the clay surface, periphyton in our study could have been exposed to nanoparticulate Ag, ionic Ag, or a mixture of both species. Therefore, in the context of our study, AgNP toxicity refers to the negative effects produced by our AgNP addition treatments instead of direct interactions between AgNPs and biota. The release of ionic Ag from AgNPs is an important mechanism for AgNP toxicity [1,41]. Ionic Ag enters algal cells via membrane transport systems and once in the cell, interferes with a variety of functions including photosynthesis, cell division, and lipid metabolism [42]. AgNPs facilitate ionic Ag uptake by acting as Ag^+^ delivery systems by releasing Ag^+^ at the cell surface [1,43], increasing ionic uptake through diffusion, or by releasing Ag^+^ after being assimilated by the cell in an intact form [38]. Given the strong potential binding of Ag^+^ with clay [35] and phosphate [22], such targeted Ag^+^ release may have been particularly important in our study. Other potential mechanisms of toxicity include the nanoparticles themselves and the creation of reactive oxygen species (ROS) [40,42]. Future studies could measure ROS in exposed periphyton to assess this possibility.

While our study design did not allow us to determine if AgNP toxicity was caused by the release of ionic Ag or by the direct interaction of AgNPs with algal cells, diffusion through porous materials is a likely exposure scenario for organisms colonizing surfaces in lakes and streams. Our study is thus an important first test of how periphyton will respond to AgNP saturated surfaces. However, we recognize that the chemistry of the porous material, of the AgNPs, and of the periphyton matrix may all have important effects on AgNP behavior and toxicity. For example, some studies have shown that algal exudates reduce AgNP toxicity by complexing with ionic Ag [44] and it is intuitive to suggest that the extracellular polymeric substances that contribute to the 3-dimensional structure of periphyton would alter AgNP toxicity. The timing of AgNP exposure may also be important for interpreting our results. In our study, algae were exposed to AgNPs during colonization, a time when the periphyton matrix is relatively undeveloped and when AgNPs may be particularly toxic [44]. Established periphyton assemblages have been shown to be less susceptible to metal toxicity, likely because thicker biofilms slow diffusion of metal ions to the inner layers [45].

We expected that P availability would lessen the negative effects of AgNP exposure on autotrophic standing stocks. Increasing P availability has been shown to decrease metal toxicity for algae [46] due to the role of intracellular phosphate bodies in metal detoxification [21,47] and the alleviation of metal-induced nutrient limitation [45]. However, P addition increased the severity of negative AgNP effects on Chl-*a* abundance in our study, particularly in L224. It is possible that high P conditions favored the formation of Ag-P complexes [22] that were assimilated by autotrophs. Such “accidental” uptake of Ag has been shown for other ligands such as thiosulfate [48,49]. However, Choi et al. [11] found that phosphate addition (up to 100 μM) had little effect on AgNP toxicity to ammonia oxidizing bacteria and McTeer et al. [24] observed more AgNO_3_-derived Ag uptake by *Chlamydomonas reinhardtii* under low P conditions compared to high P availability with no effect of P on AgNP-derived Ag uptake. The potential of P as a natural ligand for ionic Ag needs to be investigated further, particularly in natural environments.

An alternative explanation is that increasing P supply altered the composition of the autotroph community on our substrates [50]. In a previous study in these lakes, greater P-supply reduced the prevalence of diatoms and increased the relative abundance of green algae [15]. A similar shift here may have resulted in changes in biomass responses if green algae are more sensitive to AgNP toxicity. However, there is little information on the relative sensitivity of various algal taxa to AgNPs and even less about how that would mediate responses in natural environments. The taxonomic composition of algal communities (and other microbes) may have an unappreciated importance in determining the nature of AgNP toxicity in aquatic ecosystems [51].

Our results contrast with the investigation of P*AgNP interactions on natural phytoplankton assemblages by Das et al. [23], where they conducted algal growth bioassays with AgNP exposures ranging from 0–50 μg AgNP/L and phosphorus concentrations ranging from 0–100 μg-P/L. They found that AgNP exposure increased P uptake resulting in lower phytoplankton C:P ratios. They also observed P*Ag interactions on phytoplankton growth and stoichiometry, but in the opposite direction to what we found. In their study, AgNP exposure inhibited phytoplankton growth and lowered phytoplankton C:P ratios and P addition decreased the magnitude of these effects while we found that P addition enhanced the negative response of periphyton growth to AgNP exposure but compensated for the increase in periphyton C:P on AgNP-only substrates.

Direct comparisons of various AgNP studies are difficult for several reasons, including differences in the size, shape, and coatings of the AgNPs used [2]. The AgNPs used in our study were 50 nm spheres with PVP coating while Das et al. used 10 nm particles with polyacrylate coating [23]. The zeta-potential of the AgNPs also likely differed between studies, although this information was not provided by Das et al [23]. However, the contrasting results between our study and that of Das et al. suggest that AgNPs and their interactions with nutrient availability may affect the growth and stoichiometry of pelagic and benthic microbial assemblages differently. This would not be surprising given the differences in the physical structure and biomass composition of these two communities. The development of the 3-dimensional structure of periphyton may also be vulnerable to P*AgNP interactions, a dynamic that is missing in phytoplankton communities. Phytoplankton have direct access to nutrients and toxins in the water column while diffusion of these substances into the periphyton matrix is slowed by the boundary layer [52]. Periphyton nutrient cycling can be largely internal [52,53] with exoenzyme production and internal diffusion rates presumably important drivers of nutrient acquisition. Particulate and ionic Ag may interact with the extracellular polymeric substances in periphyton in various ways. Indeed, AgNPs can inhibit exoenzyme activity [54,55] but not consistently [8]. Exoenzyme production is presumably less important for nutrient uptake in planktonic communities, which may explain why phytoplankton and periphyton assemblages C:P had opposite responses to AgNP exposure and why P addition alleviated the negative AgNP effects on periphyton C:P in our study. Alternatively, AgNP exposure may have altered the periphyton biomass composition [56], with the loss of P-rich algal cells and the accumulation of nutrient-poor detritus. It is also possible that there is a non-linear response of algal growth and nutrient uptake with AgNP concentration as has been observed with other contaminants [57]. It is important to note as well that Das et al. quantified the short term (3 days) response of phytoplankton to a single pulsed AgNP addition while the organisms in our study were exposed to a continuous dose of ionic or nanoparticulate Ag diffusing from the substrates. Benthic communities likely experience AgNP exposure via both pulsed and diffusive exposure regimens. Initial exposure may occur from the overlying water column, either episodically (such as a spill or pulsed runoff) or chronically (such as continuous addition of contaminated waste water). AgNPs may also accumulate in and eventually leach from the sediments, a scenario that more closely resembles the diffusing substrates in our study.

We found that the AgNP effects on periphyton varied among the three lakes, which suggests that environmental conditions affect AgNP toxicity for freshwater organisms. The lakes differ in several aspects, but perhaps most notably in their DOC concentrations, which has been found to modify the toxicity of AgNPs in laboratory assays [58,59,60]. We found stronger effects of AgNP addition alone in the two lakes with higher levels of DOC, L239 and L222, but stronger P*Ag interactions in the most oligotrophic lake, L224. These results suggest that the effects of DOC on AgNP toxicity are more complex or different *in situ* compared to laboratory conditions. For example, DOC concentrations in our lakes were correlated with decreasing transparency. Autotrophs in L239 and L222 may have been stressed by the limited light availability, making them more susceptible to AgNP exposure. Alternatively, shade tolerant algal communities may be less tolerant to AgNP exposure.

Overall, we found that AgNP exposure can decrease periphyton growth and nutritional quality and that nutrient availability can alter the magnitude of these effects. Our results highlight the importance of *in situ* assays to determine the effects of AgNP addition to natural environments. Changes in periphyton abundance and quality likely have cascading effects on benthic and pelagic food webs. Elucidating the effects of interactions between AgNP exposure and nutrient availability is vital to understanding the impact of AgNP release to freshwater ecosystems. We suggest that future studies focus on resolving the mechanisms of AgNP toxicity relevant to structurally complex benthic communities, specifically the role of extracellular polymeric substances in AgNP retention and toxicity as well as the ways in which community composition responds to AgNP exposure in ambient and nutrient enriched conditions.

## Supporting Information

S1 Table Silver, chlorophyll, and nutrient ratios of each replicate substrate collected from all three lakes. (DOCX)
