# Supplementary material for: Phosphorus Availability Alters the Effects of Silver Nanoparticles on Periphyton Growth and Stoichiometry
Source: PLoS One. 2015 Jun 15;10(6):e0129328. doi: 10.1371/journal.pone.0129328 (PMC4468089; doi:10.1371/journal.pone.0129328)
Supplement: S1 Table — (DOCX) [file pone.0129328.s001.docx]

**Table S1. Silver, chlorophyll, and nutrient ratios of each replicate substrate collected from all three lakes.** bd indicates silver samples that were below detection and nc indicates samples that were not collected.

| **Lake** | **replicate** | **P treatment** | **Ag treatment** | **C:N (molar)** | **Ag (ng/cm^2^)** | **Chla (µg/cm^2^)** | **C:P (molar)** | **N:P (molar)** | **C:Chl** |
| --- | --- | --- | --- | --- | --- | --- | --- | --- | --- |
| L222 | A | NO P | NO Ag | 11.12 | 0.435 | 0.999 | 238.27 | 21.44 | 39.31 |
| L222 | B | NO P | NO Ag | 13.20 | 0.114 | 0.967 | 421.76 | 31.94 | 64.28 |
| L222 | C | NO P | NO Ag | 15.63 | 0.079 | 0.555 | 287.04 | 18.37 | 80.34 |
| L222 | A | NO P | LOW Ag | 10.44 | 0.308 | 1.092 | 337.50 | 32.33 | 35.98 |
| L222 | B | NO P | LOW Ag | 8.12 | 0.880 | 1.166 | 327.05 | 40.30 | 51.74 |
| L222 | C | NO P | LOW Ag | 9.17 | 1.225 | 0.845 | 258.05 | 28.13 | 57.35 |
| L222 | A | NO P | HIGH Ag | 11.47 | 0.198 | 0.348 | 275.84 | 24.05 | 87.28 |
| L222 | B | NO P | HIGH Ag | 8.61 | 4.340 | 0.514 | 283.85 | 32.98 | 50.63 |
| L222 | C | NO P | HIGH Ag | 9.64 | 0.228 | 0.400 | 437.92 | 45.41 | 106.51 |
| L222 | A | LOW P | NO Ag | 12.29 | 0.449 | 1.382 | 111.35 | 9.06 | 40.32 |
| L222 | B | LOW P | NO Ag | 9.00 | 0.066 | 0.725 | 130.36 | 14.49 | 68.15 |
| L222 | C | LOW P | NO Ag | 11.38 | 0.103 | 0.642 | 112.77 | 9.91 | 49.59 |
| L222 | A | LOW P | LOW Ag | 13.18 | 0.105 | 0.617 | 117.05 | 8.88 | 51.53 |
| L222 | B | LOW P | LOW Ag | 9.74 | 8.268 | 1.190 | 90.42 | 9.28 | 32.35 |
| L222 | C | LOW P | LOW Ag | 12.71 | 12.644 | 1.130 | 76.38 | 6.01 | 62.19 |
| L222 | A | LOW P | HIGH Ag | 11.02 | 0.773 | 0.624 | 196.31 | 17.82 | 62.11 |
| L222 | B | LOW P | HIGH Ag | 10.64 | 0.144 | 0.582 | 170.80 | 16.06 | 68.79 |
| L222 | C | LOW P | HIGH Ag | 13.00 | 0.092 | 0.478 | 190.61 | 14.67 | 66.70 |
| L222 | A | HIGH P | NO Ag | 11.97 | 0.182 | 1.270 | 111.73 | 9.33 | 46.47 |
| L222 | B | HIGH P | NO Ag | 13.18 | 0.111 | 1.234 | 99.54 | 7.55 | 33.83 |
| L222 | C | HIGH P | NO Ag | 11.18 | 0.035 | 1.105 | 143.45 | 12.83 | 55.87 |
| L222 | A | HIGH P | LOW Ag | 12.23 | 4.753 | 1.000 | 77.62 | 6.35 | 41.23 |
| L222 | B | HIGH P | LOW Ag | 11.85 | 0.538 | 0.410 | 99.15 | 8.37 | 57.23 |
| L222 | C | HIGH P | LOW Ag | 7.76 | 0.257 | 1.373 | 161.65 | 20.83 | 37.90 |
| L222 | A | HIGH P | HIGH Ag | 14.95 | 0.725 | 0.180 | 252.08 | 16.86 | 202.07 |
| L222 | B | HIGH P | HIGH Ag | 17.69 | 1.613 | 0.133 | 256.81 | 14.52 | 243.08 |
| L222 | C | HIGH P | HIGH Ag | 10.30 | 0.285 | 0.114 | 187.43 | 18.20 | 182.22 |
| L224 | A | NO P | NO Ag | 9.35 | 0.005 | 0.591 | 202.76 | 21.70 | 71.87 |
| L224 | B | NO P | NO Ag | 7.72 | 0.020 | 0.701 | 176.74 | 22.91 | 65.38 |
| L224 | C | NO P | NO Ag | 13.28 | 0.010 | 0.116 | 171.21 | 12.89 | 278.20 |
| L224 | A | NO P | LOW Ag | 7.06 | 0.153 | 0.664 | 243.39 | 34.49 | 75.56 |
| L224 | B | NO P | LOW Ag | 9.70 | 0.817 | 0.524 | 526.22 | 54.23 | 87.24 |
| L224 | C | NO P | LOW Ag | 8.71 | 0.080 | 0.525 | 339.22 | 38.95 | 95.99 |
| L224 | A | NO P | HIGH Ag | 11.18 | 0.022 | 0.367 | 291.51 | 26.08 | 122.71 |
| L224 | B | NO P | HIGH Ag | 9.11 | 0.033 | 0.581 | 398.13 | 43.71 | 90.91 |
| L224 | C | NO P | HIGH Ag | 8.14 | 2.138 | 0.361 | 432.21 | 53.08 | 142.27 |
| L224 | A | LOW P | NO Ag | 7.72 | 0.040 | 1.262 | 140.42 | 18.19 | 41.43 |
| L224 | B | LOW P | NO Ag | 9.15 | bd | 0.603 | 70.07 | 7.66 | 59.77 |
| L224 | C | LOW P | NO Ag | 7.09 | 0.022 | 0.819 | 62.84 | 8.86 | 57.19 |
| L224 | A | LOW P | LOW Ag | 9.37 | 0.085 | 0.774 | 65.08 | 6.95 | 52.23 |
| L224 | B | LOW P | LOW Ag | 10.13 | bd | 0.867 | 105.19 | 10.38 | 52.42 |
| L224 | C | LOW P | LOW Ag | 9.70 | 0.391 | 0.967 | 138.64 | 14.30 | 54.59 |
| L224 | A | LOW P | HIGH Ag | 6.91 | 0.050 | 0.626 | 109.94 | 15.91 | 70.71 |
| L224 | B | LOW P | HIGH Ag | 8.74 | 0.017 | 0.618 | 172.73 | 19.76 | 71.73 |
| L224 | C | LOW P | HIGH Ag | 7.26 | 0.469 | 0.806 | 133.07 | 18.33 | 68.48 |
| L224 | A | HIGH P | NO Ag | 7.96 | 0.039 | 1.419 | 139.52 | 17.54 | 46.45 |
| L224 | B | HIGH P | NO Ag | 10.48 | 0.059 | 1.431 | 64.25 | 6.13 | 48.94 |
| L224 | C | HIGH P | NO Ag | 8.29 | bd | 1.311 | 62.54 | 7.55 | 47.11 |
| L224 | A | HIGH P | LOW Ag | 7.62 | 0.256 | 0.397 | 62.96 | 8.26 | 107.61 |
| L224 | B | HIGH P | LOW Ag | 11.56 | 0.549 | 0.866 | 69.69 | 6.03 | 52.78 |
| L224 | C | HIGH P | LOW Ag | 10.66 | 0.016 | 0.663 | 66.74 | 6.26 | 71.08 |
| L224 | A | HIGH P | HIGH Ag | 13.53 | 0.499 | 0.141 | 235.07 | 17.37 | 189.13 |
| L224 | B | HIGH P | HIGH Ag | 13.46 | 0.493 | 0.115 | 215.88 | 16.03 | 268.27 |
| L224 | C | HIGH P | HIGH Ag | 9.97 | 0.122 | 0.027 | 239.13 | 23.99 | 833.21 |
| L239 | A | NO P | NO Ag | 7.54 | nc | 0.988 | 228.94 | 30.38 | 89.63 |
| L239 | B | NO P | NO Ag | 10.24 | 0.054 | 0.602 | 182.95 | 17.86 | 111.33 |
| L239 | C | NO P | NO Ag | 10.89 | 0.016 | 0.358 | 385.87 | 35.43 | 182.07 |
| L239 | A | NO P | LOW Ag | 12.22 | 0.138 | 0.481 | 276.53 | 22.62 | 116.01 |
| L239 | B | NO P | LOW Ag | 11.46 | 0.037 | 0.129 | 856.33 | 74.70 | 270.22 |
| L239 | C | NO P | LOW Ag | 9.99 | 0.167 | 0.607 | 203.11 | 20.33 | 123.27 |
| L239 | A | NO P | HIGH Ag | 10.60 | 0.030 | 0.578 | 373.79 | 35.25 | 137.88 |
| L239 | B | NO P | HIGH Ag | 17.52 | nc | 0.346 | 409.92 | 23.40 | 110.40 |
| L239 | C | NO P | HIGH Ag | 13.36 | 7.870 | 0.490 | 195.54 | 14.64 | 123.48 |
| L239 | A | LOW P | NO Ag | 8.13 | bd | 1.504 | 115.53 | 14.21 | 48.57 |
| L239 | B | LOW P | NO Ag | 7.15 | 0.024 | 0.819 | 98.60 | 13.79 | 89.58 |
| L239 | C | LOW P | NO Ag | 10.24 | 0.019 | 0.944 | 87.78 | 8.57 | 88.38 |
| L239 | A | LOW P | LOW Ag | 10.62 | 0.157 | 1.140 | 95.76 | 9.02 | 55.91 |
| L239 | B | LOW P | LOW Ag | 6.50 | 0.030 | 0.879 | 286.59 | 44.10 | 66.96 |
| L239 | C | LOW P | LOW Ag | 9.96 | 0.013 | 1.106 | 109.01 | 10.95 | 64.96 |
| L239 | A | LOW P | HIGH Ag | 10.07 | 0.581 | 0.678 | 190.14 | 18.88 | 129.20 |
| L239 | B | LOW P | HIGH Ag | 11.57 | 0.198 | 0.541 | 170.17 | 14.71 | 138.72 |
| L239 | C | LOW P | HIGH Ag | 12.40 | 0.090 | 0.138 | 252.65 | 20.38 | 465.69 |
| L239 | A | HIGH P | NO Ag | 11.71 | bd | 1.385 | 89.62 | 7.65 | 57.98 |
| L239 | B | HIGH P | NO Ag | 13.50 | bd | 0.120 | 366.56 | 27.15 | 618.83 |
| L239 | C | HIGH P | NO Ag | 10.31 | bd | 0.956 | 129.12 | 12.53 | 123.57 |
| L239 | A | HIGH P | LOW Ag | 14.37 | 0.025 | 0.805 | 85.97 | 5.98 | 83.94 |
| L239 | B | HIGH P | LOW Ag | 10.65 | 0.015 | 1.160 | 72.08 | 6.77 | 54.16 |
| L239 | C | HIGH P | LOW Ag | 14.31 | bd | 0.615 | 97.81 | 6.84 | 90.49 |
| L239 | A | HIGH P | HIGH Ag | nc | 0.038 | 0.199 | nc | nc | nc |
| L239 | B | HIGH P | HIGH Ag | 7.91 | 0.076 | 0.248 | 67.15 | 9.90 | 164.02 |
| L239 | C | HIGH P | HIGH Ag | 20.36 | nc | 0.025 | 40.22 | 2.30 | 793.92 |
